# L-shaped association of serum α-Klotho and frailty among the middle-aged and older adults: results from NHANES 2007–2016

**DOI:** 10.1186/s12877-023-04324-z

**Published:** 2023-11-03

**Authors:** Zewei Jiang, Jiaxin Wang, Xingdong Cai, Ping Wang, Shengming Liu

**Affiliations:** 1 Department of Pulmonary and Critical Care Medicine, The First Affiliated Hospital of Jinan University, Guangzhou, 510630 China; 2 Department of Endocrinology and Metabolism, The First Affiliated Hospital of Jinan University, Guangzhou, 510630 China; 3 Emergency Department, Zhujiang Hospital, Southern Medical University, Guangzhou, 510280 China

**Keywords:** Frailty, α-Klotho, Middle-aged and older adults, NHANES

## Abstract

**Background:**

Frailty is common and not limited to older age group. Serum α-Klotho works as a biomarker of anti-aging effect. However, there is limited research about the relationship between them in middle-aged and older people and controversy still exists.

**Methods:**

Based on data from National Health and Nutrition Examination Survey (NHANES) 2007–2016, we constructed weighted logistic regression models and conducted sensitivity tests to investigate the correlation between frailty and α-Klotho among people aged 40 to 79. And then their relationship was visualized by Restricted Cubic Spline (RCS). Finally, the stratified analyses and interaction tests of covariables was presented in the forest plot.

**Results:**

A total of 7052 individuals were involved in this study, with mean age of 62.76 ± 0.18 years and females accounting for 51.05%. 2554 of them were in “frailty”. After adjustment for relevant covariables, weighted logistic regression models showed that the odds ratio and 95% confidence interval [ORs (95%CI)] of correlation between frailty and Natural Logarithm(ln)-transformed α- Klotho[ln(α-Klotho)] was 0.63 (0.50, 0.79); we then performed a sensitivity analysis and found that the results remained stable. In model 3, individuals in quartiles 2, 3, and 4 showed statistical differences compared with the lowest ln(α-Klotho) quartiles, ORs (95% CI) were 0.74 (0.59, 0.93), 0.72 (0.57, 0.91), 0.71 (0.57, 0.87), respectively. Subsequently, non-linear associations were exhibited by RCS (*p*<0.001). The turning point for α-Klotho and ln(α-Klotho) were 785.7(pg/ml) and 6.67, respectively. Finally, analysis of the relationship between different levels of ln(α-Klotho) and frailty in different populations revealed differences between groups. The results of the interaction test showed that no other covariables had significant interaction with serum α-Klotho in our study.

**Conclusion:**

The L-shaped and negative correlation was found between α-Klotho and frailty among people aged 40 to 79 in the NHANES from 2007 to 2016.

**Supplementary Information:**

The online version contains supplementary material available at 10.1186/s12877-023-04324-z.

## Introduction

Frailty is a multifactorial syndrome with dysfunction of physiological systems that increases with age and decreases survival rates at any age [1]. It has been exposed as the most problematic manifestation of ageing [2], which increases vulnerability of people to stressors [3] and the incidence of undesirable events, such as fall, disability and hospital admission [4–6]. Frailty is commonly found in patients who are in the intensive care unit (ICU) [7] and has become a growing global health problem [8]. However, frailty assessment criteria have not been standardized. Two common approaches are always applied to describe this state: frailty phenotype [4, 9] and the model of frailty index (FI) [9]. Besides the ways mentioned above, other methods included simplified frailty phenotype and prognostic frailty score [10]. The assessment of frailty phenotype includes a combination of five aspects: shrinking, weakness, poor endurance and energy, slowness, low physical activity level [4]. Compared with the frailty phenotype, FI performs better at distinguishing status of frailty [11]. The counts and items about FI were inconsistent in several different studies [11–13]. In our analysis, based on the reliable and obtainable clinical features from the examination data, laboratory data, and questionnaire data of NHANES dataset, a frailty index with 49 items was used for later analyzing [14–16].

The human klotho gene, located at the chromosome 13q12 and consisting of 5 exons [17], encoded two forms of protein: membrane bound and circulating forms [17, 18]. α-Klotho was the form following cleavage from transmembrane form [19], which was identified to include three forms that may have diverse functions, such as full-length transmembrane, truncated soluble and secreted form [20]. The easiest measuring form is plasma Klotho [5]. Soluble α-Klotho is a multifunctional protein [20] and associated with anti-oxidative and anti-inflammatory [21]. Previous researches have illustrated that serum Klotho was associated with chronic kidney disease [22], cardiovascular disease [23] and diabetes [24].

Critical focus on frailty screening is needed in clinical practice [25, 26], because not all the older suffer from frailty [27, 28]. The expression of Klotho decreases after 40 years old [5]. The relationship between the incidence of frailty and α-Klotho remains ambiguous. In order to fill the gap, large-scale studies focusing on middle-aged and older population with their serum α-Klotho protein and frailty are urgently needed. We expect to search for indicators that can identify the status of frailty and prevent or reduce the incidence of adverse events through early interventions, which is essential to reduce the burden on patients, families, and society.

## Methods

### Study design

cross-sectional study.

### Sample population

**Fig. 1:**
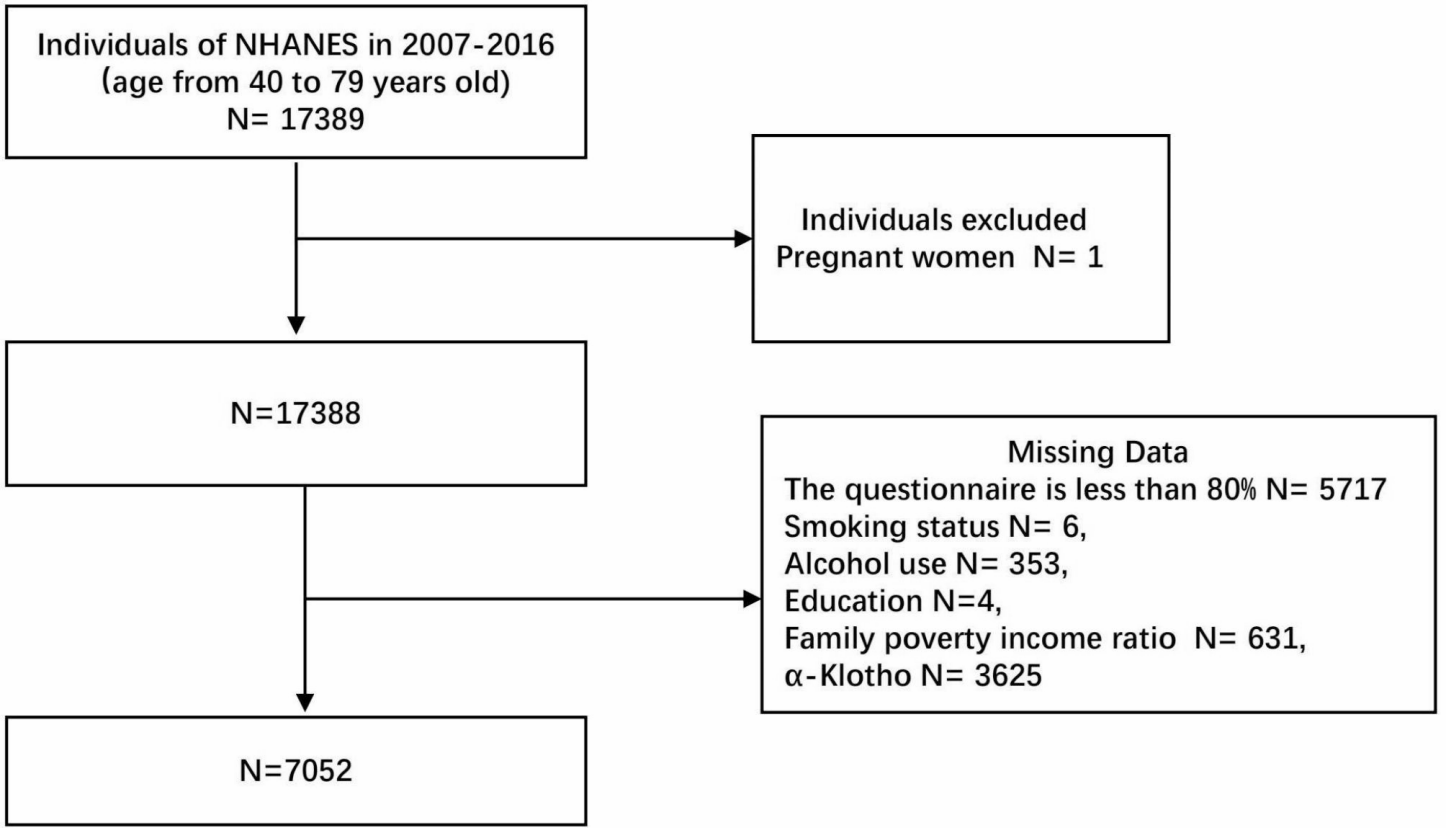
Flow chart of screening

The data from NHANES is updated every 2 years. The methodological design of a complex, stratified, multi-stage, probability sample was used for gathering data of targeted groups. Five continuous cycles (2007–2008, 2009–2010, 2011–2012, 2013–2014, 2015–2016) were involved in our investigation, which included 17,389 participants. All the participants gave handwritten agreement and passed the ethics reviews from the National Center for Health Statistics (NCHS) Ethics Review Board. Serum α-Klotho protein was only measured at the ages 40 to 79 years in the NHANES dataset. We choose this interval age and exclude individuals with pregnancy or without complete data. Finally, 7052 participants met the screening requirements and were enrolled in analysis. Details about the screening process were shown in Fig. 1. Other survey methods can be traced at NHANES websites (https://www.cdc.gov/nchs/nhanes/index.htm).

### Serum α-Klotho protein

Clinical samples available were collected by specialist workers on dry ice and stored at -80 ℃. Samples of participants were tested by enzyme-linked immunosorbent assay (ELISA). Each sample was assayed in duplicate. The mean value of two samples was calculated as the final result. If two outcomes exceeded 10%, the procedure would be repeated. The criteria of laboratory quality control and monitoring can be traced at the NHANES websites [29, 30].

### Assessment of frailty

The eligibility questionnaire requires at least 80% completion of 49 frailty items. It has been reported that if sufficient number of variables are included (about 40), they can be randomly selected and still give similar results for risk of poor outcome [13, 31]. Handgrip strength was not included in this frailty index model because not all five selection cycles had data of handgrip strength. As data was available from the NHANES dataset, one item (difficulty with leisure activities at home) was included in relation to the literature reporting that frailty is associated with physical activity. We included this and the remaining 48 items to form a 49-item frailty index model including 7 main categories: cognition (1 item), dependence (16 items), depressive conditions (7 items), comorbidities (13 items), hospital and care (5 items), physical anthropometry (1 item), and laboratory results (6 items), the details of which were presented in the Supplementary Table 1 [14–16]. The scores for the specific frailty items are summed up and subsequently divided by the amount of frailty items. The severity of the deficit is assessed to give a number between 0 and 1. Number 0 stands for no deficit and 1 for a full deficit. This method allows us to transform continuous to categorical variables for the next analysis. As for the frailty index, we classified it into dichotomized status (≤ 0.21, > 0.21). The cutoff value was based on previous literature [14].

### Covariates

Sex was divided into male and female. Race/ethnicity was categorized to 4 subgroups: Non-Hispanic White, Non-Hispanic Black, Mexican American, and Other. Education levels were subcategorized into three levels: less than high school, high school or its equivalent, and more than high school. Smoking status were grouped into three categories: never (smoked less than 100 cigarettes in life), former (smoked more than 100 cigarettes in life and smoke not at all now), now (smoked more than 100 cigarettes in life and smoke some days or every day). Alcohol use was classified into five groups according the definition of questionnaire (last year): never (fewer than 12 drinks in lifetime), mild (≤ 2 drinks every day for males, ≤ 1 drinks every day for female), moderate (≤ 3 and > 2 drinks every day for males, ≤ 2 and > 1 drinks every day for female or ≥ 2 to < 5 days per month of binge drinking), heavy (≥ 4 drinks every day for males, ≥ 3 drinks every day for female or ≥ 5 days per month of binge drinking), former (did not drink last year, but had ≥ 12 drinks in 1 year or drank ≥ 12 drinks in lifetime). Frailty was divided into two groups with non-frailty (≤ 0.21) or frailty (>0.21) [14]. Ratio of family income to poverty was divided into three groups (< 130%, 130–350%, ≥ 350%) [32].

### Statistical analysis

The statistical analyses in our study were carried out with weights. The α-Klotho was categorized into quartiles (Q, pg/ml), which are Q1(≤ 641.775), Q2 (641.775, 785.7), Q3 (785.7, 973.9) and Q4 (> 973.9), respectively. The Chi-square (χ^2^) test was employed for categorical variables and one-way Analysis of Variance (ANOVA) test for continuous variables, which were used to search for differences between α-Klotho quartiles. The results of continuous variables were reported as mean ± standard deviation (SD). For binary or multi-categorical variables, frequency counts and proportions were presented in Table 1. The serum α-Klotho concentration was calculated using the ln-transformation due to the skewed distribution. To explore the linkage between the frailty and ln(α-Klotho), three weighted logistic regression models were constructed. No covariables were adjusted in Model 1. Model 2 was adjusted for sex, age, and race/ethnicity and model 3 for all covariates from Table 1 except frailty and number. Sensitivity analysis was performed to assess whether the results were stable. And then their associations were visualized by using RCS. Finally, subgroup analysis was performed to detect the association of ln(α-Klotho) and frailty between different populations. Interaction test between ln(α-Klotho) and frailty was assessed among the middle-aged and older adults from NHANES 2007–2016. R software 4.2.2 was carried out in this study. Statistical significance is defined as a two-tailed *p*-value threshold of less than 0.05.

**Table 1:** Characteristic of the study population in NHANES 2007–2016

Variables	Total		Q1	Q2	Q3	Q4	*P* value
**N (%)**	7052 (100)		1763 (25.00)	1764 (25.01)	1762 (24.99)	1763 (25.00)	
**Age (years)**	62.76 ± 0.18		63.92 ± 0.29	62.43 ± 0.32	62.61 ± 0.30	62.09 ± 0.33	< 0.0001*
**Sex (%)**							0.01*
Female	3600 (51.05)		852 (51.87)	833 (49.59)	928 (54.55)	987 (57.67)	
Male	3452 (48.95)		911 (48.13)	931 (50.41)	834 (45.45)	776 (42.33)	
**Ethnicity/Race (%)**							0.003*
Non-Hispanic White	3378 (47.90)		875 (77.71)	904 (79.41)	858 (77.76)	741 (73.98)	
Non-Hispanic Black	1442 (20.45)		370 (9.19)	321 (7.51)	307 (7.33)	444 (11.21)	
Mexican American	952 (13.50)		238 (4.53)	232 (4.41)	243 (4.96)	239 (5.06)	
Other	1280 (18.15)		280 (8.58)	307 (8.67)	354 (9.95)	339 (9.75)	
**Education (%)**							0.04*
Less than high school	1020 (14.46)		254 (6.53)	260 (6.35)	248 (6.68)	258 (7.32)	
High school or equivalent	2737 (38.81)		728 (39.37)	658 (33.75)	684 (33.79)	667 (33.43)	
College or above	3295 (46.72)		781 (54.10)	846 (59.90)	830 (59.54)	838 (59.25)	
**Smoke Status (%)**							0.04*
Never	3192 (45.26)		725 (41.47)	757 (44.19)	831 (46.18)	879 (48.78)	
Former	2464 (34.94)		661 (38.84)	646 (37.79)	602 (35.79)	555 (32.12)	
Now	1396 (19.80)		377 (19.69)	361 (18.02)	329 (18.03)	329 (19.10)	
**Alcohol Use (%)**							0.004*
Never	1105 (15.67)		220 (9.26)	285 (13.41)	275 (11.59)	325 (13.73)	
Mild	2474 (35.08)		610 (39.93)	607 (40.00)	651 (41.68)	606 (42.85)	
Moderate	810 (11.49)		224 (14.94)	207 (13.30)	199 (13.51)	180 (11.10)	
Heavy	797 (11.30)		219 (11.47)	216 (12.37)	200 (11.84)	162 (7.66)	
Former	1866 (26.46)		490 (24.40)	449 (20.91)	437 (21.38)	490 (24.64)	
**Frailty (%)**							0.004*
Non-Frailty	4498 (63.78)		1017 (63.86)	1155 (70.68)	1188 (70.77)	1138 (69.08)	
Frailty	2554 (36.22)		746 (36.14)	609 (29.32)	574 (29.23)	625 (30.92)	
**Family income to poverty ratio (%)**							0.5
<1.3	2487 (35.27)		627 (21.80)	631 (21.99)	611 (21.24)	618 (23.63)	
1.3–3.5	2623 (37.20)		661 (37.55)	627 (34.55)	659 (36.57)	676 (36.88)	
≥3.5	1942 (27.54)		475 (40.64)	506 (43.46)	492 (42.19)	469 (39.49)	

Note 1: N: number; Q1 (≤ 641.775), Q2 (641.775, 785.7), Q3 (785.7, 973.9), Q4 (> 973.9); * significantly difference

## Results

### The baseline features of the participants

7052 individuals were enrolled in our study. The average age was 62.76 ± 0.18 years and 51.05% were female. Classified in accordance with the frailty index used in this study, 36.22% of the participants were in “frailty”. The weighted sampling design represents 50,988,947 non- institutionalized residents in the United States. The analysis highlights statistical differences in age, sex, race/ethnicity, education level, smoke status, alcohol use and frailty between groups in different concentrations of α-Klotho. No significant differences were observed in the ratio of family income to poverty. The general characteristics of this study population are listed in Table 1.

### The relationship between ln(α-Klotho) and frailty

**Fig. 2:**
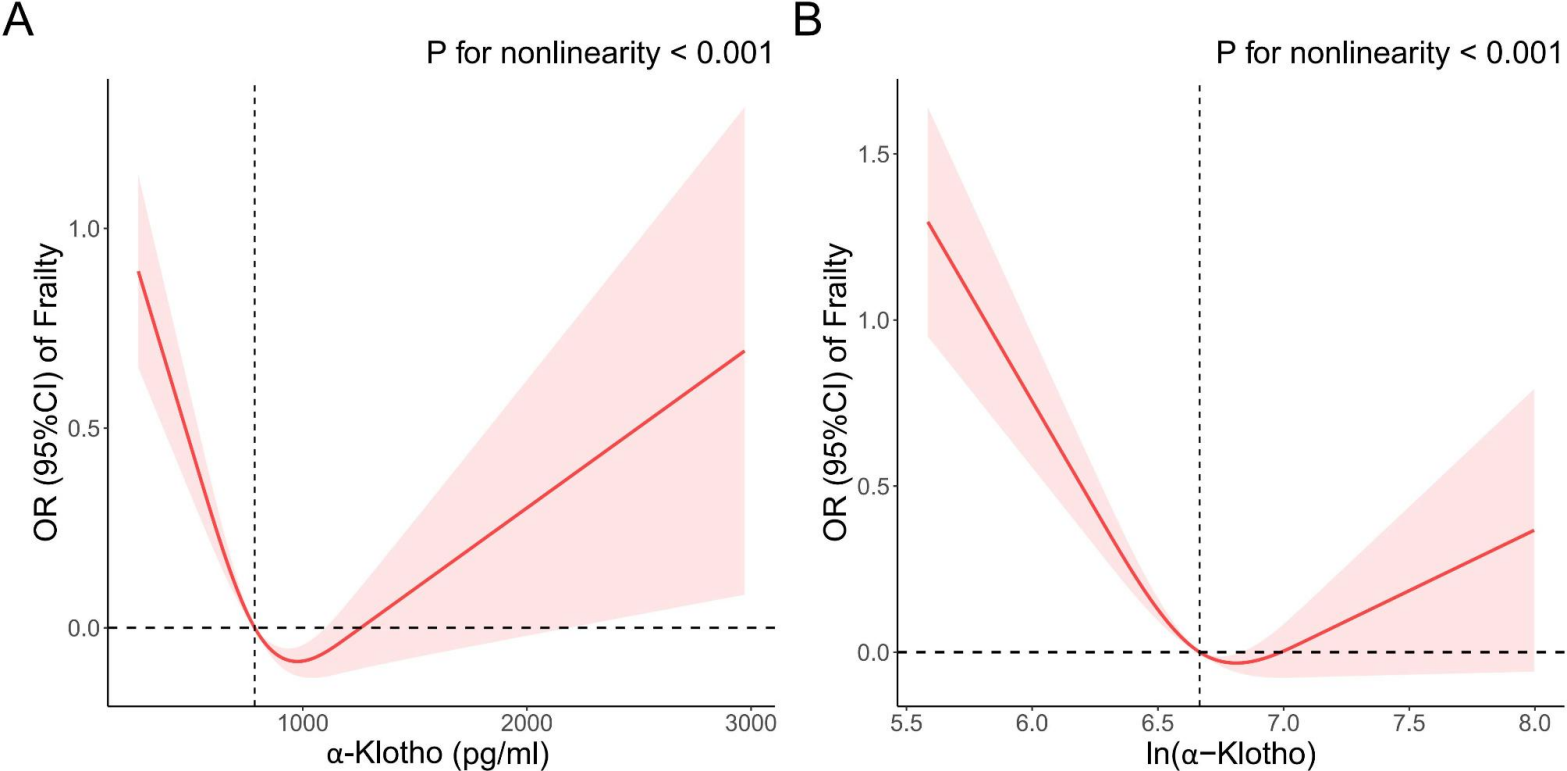
RCS analysis with multivariate-adjusted associations between frailty and α-Klotho, ln(α-Klotho)

Three weighted logistic regression models were fitted to explore the correlation between ln(α-Klotho) and frailty, as shown in Table 2. The ORs (95% CI) were 0.73 (0.59, 0.90), 0.63 (0.51, 0.79), 0.63 (0.50, 0.79) respectively in model 1, model 2 and model 3. Sensitivity analysis transform the ln(α-Klotho) into quartile variables, which are Q1 (≤ 6.464), Q2 (6.464, 6.667), Q3 (6.667, 6.881), and Q4 (> 6.881). Adjusting for potential variables or not, the relationship between ln(α-Klotho) quartiles and frailty in all 3 models are in line with the continuous variable. In model 3, compared to Q1, the ORs (95%CI) was 0.74 (0.59, 0.93), 0.72 (0.57, 0.91), 0.71 (0.57, 0.87), in the participants with concentration in Q2, Q3 and Q4, respectively. RCS showed L-shaped association between frailty and α-Klotho (continuous and ln-transformed variable) in Fig. 2. The turning point for α-Klotho and ln(α-Klotho) were 785.7(pg/ml) and 6.67, respectively.

### Stratified analyses and interaction test

**Fig. 3:**
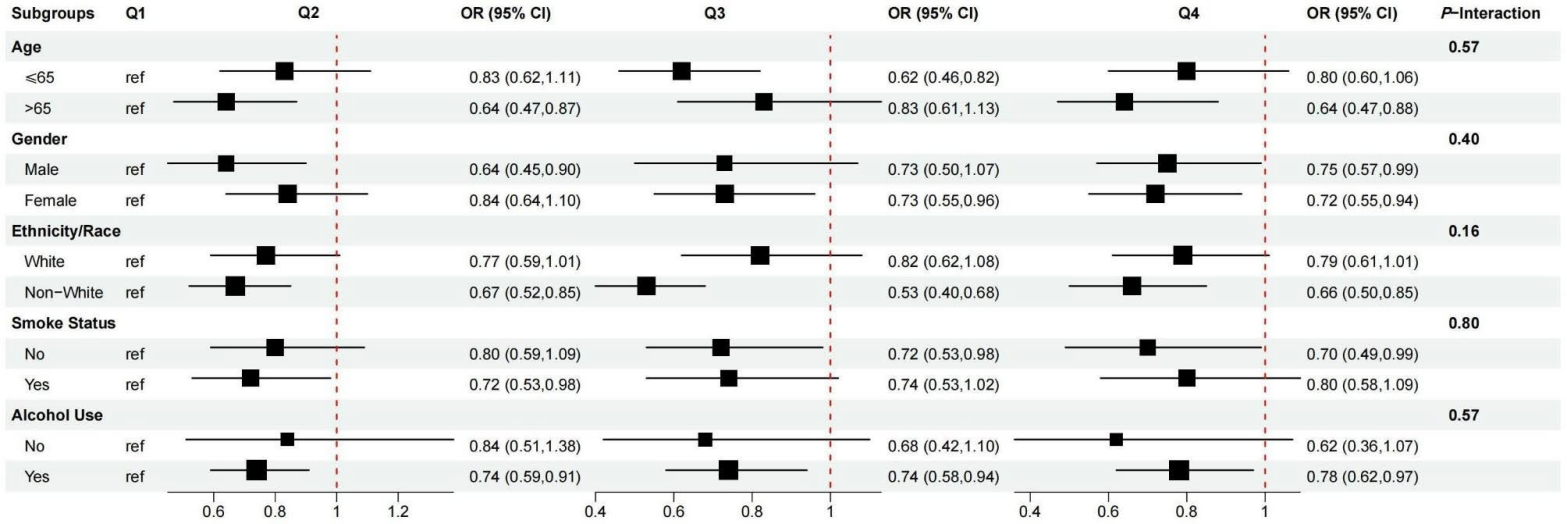
Subgroup analysis and interaction of the association between ln(α-Klotho) and frailty

After adjusting the relevant covariates (all covariates from Table 1 except frailty and number), we analyze the interaction and subgroup analysis between ln(α-Klotho) and frailty. There was no significant interaction in the subgroup analysis stratified by age, sex, race/ethnicity, smoking status, alcohol use. Subgroup analyses explored the relationship between ln(α-klotho) and frailty in different populations. Results in Fig. 3 showed that in most subgroups, ln(α-klotho) with a significant negative association with frailty, however, this relationship was not obvious in White and no alcohol use participants. Detail results of interaction and subgroups analysis were visualized in forest plot (Fig. 3).

## Discussion

7052 individuals were involved in this cross-sectional analysis, representing 50,988,947 non-institutionalized residents in the United States. The ln(α-Klotho) was negative and L-shaped associated with frailty based on the data from 5 cycles (2007–2016) of individuals from NHANES. Therefore, increasing the concentration of α-Klotho may be a potential and effective approach to improve the frailty status of the population according to the results of our study and literature review.

**Table 2:** Association between ln(α-Klotho) and frailty

	Model 1			Model 2			Model 3	
	ORs (95%CI)	*P*-value		ORs (95%CI)	*P*-value		ORs (95%CI)	*P*-value
**ln(α-Klotho)**	0.73 (0.59, 0.90)	0.005*		0.63 (0.51, 0.79)	< 0.001*		0.63 (0.50, 0.79)	< 0.001*
**Total**								
Q1	1 (ref)			1 (ref)			1 (ref)	
Q2	0.73 (0.60, 0.89)	0.002*		0.71 (0.58, 0.87)	0.001*		0.74 (0.59, 0.93)	0.01*
Q3	0.73 (0.59, 0.90)	0.004*		0.69 (0.56, 0.86)	0.001*		0.72 (0.57, 0.91)	0.01*
Q4	0.79 (0.66, 0.95)	0.01*		0.71 (0.58, 0.86)	< 0.001*		0.71 (0.57, 0.87)	0.002*
*P* for Trend		0.01*			< 0.001*			< 0.001*

Note 2: ORs = odds ratio; CI = confidence interval; Q1 (≤ 6.464), Q2 (6.464, 6.667), Q3 (6.667, 6.881), Q4 (> 6.881); No covariable was adjusted in Model 1. Model 2 was adjusted for sex, age, and race/ethnicity and Model 3 for all covariates from Table 1 except frailty and number

Frailty is an emerging global health burden and public health challenge [8, 26]. There is heterogeneity in clinical manifestations of frailty [26], and there is currently no consistent gold standard for frailty [8]. As people age, the tendency of developing comorbidities also increases. Chronic diseases have been identified as dominant determinant of frailty [33], and cognitive function is closely related to physical frailty [34]. Frailty is a dynamic state that can worse or alleviate under different conditions. It may be influenced through the treatment of chronic disease [33]. Two common approaches are always applied to describe this state: frailty phenotype [4, 9] and the model of frailty index (FI) [9]. A recent study has demonstrated that frailty index exhibits superior reproducibility and responsiveness compared to the frailty phenotype in older people who are acutely hospitalized [35].The frailty index model was developed as a tool of quantifying the frailty conditions on the basis of a comprehensive assessment.

Serum α-Klotho is possibly a biomarker for improvements in frailty. The decreasing stem cells and increasing progenitor senescence were observed in the tissues and organs of klotho mice [36]. Mutation in the mouse klotho gene cause an aging-like syndrome [37]. Whereas overexpression of klotho gene extends life spans in mice [38]. What comes as a pleasant surprise is that klotho can promotes recovery in acute kidney injury of mice [39]. It has been shown that injecting klotho protein into aging monkeys can improve their cognitive function [40]. In the reported clinical study, α-Klotho was negatively correlated with mortality among American adults [21]. The same conclusion can be seen in the study from Italy [41].

High levels of plasma Klotho were associated with a low likelihood of the frailty, in particular with exhaustion, in a study of 774 participants in the Invecchiare in Chianti, “Aging in Chianti” (InCHIANTI) [42]. However, another research of 89 individuals found no significant association between them [43]. The relationship between α-Klotho and frailty had yielded inconsistent results in previous study. This may be attributed to insufficient samples. In another study based on the NHANES database, frailty index of 34 health items and frailty phenotypes of 5 items were established to assess the relationship between frailty and klotho protein, and the literature showed that higher klotho concentration were associated with lower frailty in middle-aged and older people [44]. The same relationship can be seen in our study. We revealed the association in the real world, the conclusions of which were mutual verification with previous study. These results are robust. In addition, another prominent finding in our results that distinguishes us from the above paper is that changes in serum α-klotho protein concentration are L-shaped in association with frailty, and there is a tendency to stage changes. Although, with the increasing of α-Klotho and ln(α-Klotho), the odds of frailty decreased, when they reached threshold value (785.7 pg/ml and 6.67), the effectiveness of protection has declined to a certain extent. At present, the mechanism of this phenomenon is not clear but may closely related to oxidative stress and inflammation [45, 46]. A study has observed a phenomenon that exposure to cigarette smoke increases oxidative stress [47]. Verde Z et al. had found it in the plasma of smokers and non-smokers, the levels of Klotho were significantly higher in heavy smokers (≥ 20 pack years smoked) than in light smokers (< 20 pack years smoked) [48]. Therefore, we hypothesize that α-Klotho protein, as a component of the antioxidant defense system and an inhibitor of inflammatory damage, may exert a protective effect by reducing the detrimental effects of free radicals and reactive oxygen species on the body. Moreover, it is plausible that the upregulation of α-Klotho protein expression could serve as a compensatory response to counteract oxidative stress within the body. Furthermore, the increased expression of antioxidants correlates with elevated oxidative stress levels within the body. Under conditions of heightened levels of both antioxidants and pro-oxidants, the efficacy of antioxidants in providing protection may be compromised due to the potential damage caused by pro-oxidant component, without yielding a corresponding increase in protective effects. Nevertheless, increasing the concentration of klotho protein still helps to reduce the odds of frailty.

The strength of our study is illustrated as follows: firstly, the sample was collected from the nationally representative cohort and the sample size in this study was increased to 7052. Secondly: in terms of the description of frailty, 49 items encompass a greater number of items and provides a more detailed and comprehensive account, incorporating more specific indicators that offer deeper insights. Thirdly, sensitivity analysis showing that the results of the association between two variables remain stable, manifest the results robust in present investigation. Therefore, assessing complex frailty models with relatively simple and easily detectable intervenable indicators can reduce clinical workload and facilitate clinical implementation. The outcomes can potentially be expanded to clinical practice.

There are still some limitations in this study. On the one hand, the participants in NHANES may not represent the global population. Whether it might suit different ethnicity is less certain. Additional samples of multiple centers are needed to increase credibility. On the other hand, the data from cross-sectional study only illustrated their association. However, the causal-effect relation has not been determined.

## Conclusion

L-shaped and negative correlation was presented between α-Klotho and frailty among 40 to 79 years old in the NHANES. Our investigation provides an effective and reliable reference to optimize public and clinical health care.

### Electronic supplementary material

Below is the link to the electronic supplementary material.

Supplementary Material 1

## Data Availability

Dataset can be found at NHANES online website: https://www.cdc.gov/nchs/nhanes/index.htm.

## Abbreviations

95%CI: 95% Confidence Interval
ANOVA: Analysis of Variance
ELISA: Enzyme-linked immunosorbent assay
FI: Frailty Index
ICU: Intensive care unit
InCHIANTI: Invecchiare in Chianti, “Aging in Chianti”
ln: Natural Logarithm
N: Number
NCHS: National Center for Health Statistics
NHANES: National Health and Nutrition Examination Survey
ORs: Odds Ratio
Q: Quartiles
RCS: Restricted Cubic Spline
SD: Standard Deviation
χ2: Chi-square
